# A Clinical Manual of Skin Diseases

**Published:** 1898-11

**Authors:** 


A CLINICAL MANUAL OF SKIN DISEASES. With Special Reference to Diagnosis and Treatment, for the Use of Students and General Practitioners. By W. A. HARDAWAY, A. M., M. D., Professor of the Diseases of the Skin and Syphilis in the Missouri Medical College, St. Louis; Physician for the Diseases of the Skin to the Martha Parsons' Hospital for Children and to St. John's Hospital; Ex-President of the American Dermatological Association. Second edition, revised and enlarged, with 42 engravings and 2 plates. Philadelphia and New York: Lea Brothers & Co. 1898.

This excellent manual has undergone a thorough revision and appears in its second edition. Numerous alterations and additions are found everywhere throughout the book, the most important rearrangement being the presentation of the various diseases of the skin under a scientific system of classification, instead of alphabetically as at first. Among other changes noted is the omission of the appendices of formulæ, since the necessary directions are now found incorporated in the text. However, the general plan of the book remains the same.

As a handbook this manual is worthy of the highest praise, being a valuable and concise résumé of modern dermatology as it actually exists, rather than a digest of dermatological speculations. It embodies the recent views and advances made in pathology, closeness and accuracy in diagnosis and practical usefulness in treatment.

The work is quite fully and carefully illustrated with a few new additions which are all good and well selected. Professor Hardaway's work fulfills well its purpose as a compact, reliable and satisfactory manual, designed for students and general practitioners of medicine.

